# Experimental Investigation on Adaptive Robust Controller Designs Applied to Constrained Manipulators

**DOI:** 10.3390/s130405181

**Published:** 2013-04-18

**Authors:** Samuel L. Nogueira, Tatiana F. P. A. T. Pazelli, Adriano A. G. Siqueira, Marco H. Terra

**Affiliations:** 1 Department of Mechanical Engineering, University of São Paulo, 400 Trabalhador São-Carlense av., 13566-590, São Carlos, Brazil; E-Mail: siqueira@sc.usp.br; 2 Department of Electrical Engineering, Federal University of São Carlos,Washington Luís, km 235-SP-310, São Carlos, Brazil; E-Mail: tatianapazelli@ufscar.br; 3 Department of Electrical Engineering, University of São Paulo, 400 Trabalhador São-Carlense av., 13566-590, São Carlos, Brazil; E-Mail: terra@sc.usp.br

**Keywords:** constrained manipulators, *ℋ*_∞_ control, neural networks, fuzzy systems, variable structure control, load cell, multi-axis force sensor

## Abstract

In this paper, two interlaced studies are presented. The first is directed to the design and construction of a dynamic 3D force/moment sensor. The device is applied to provide a feedback signal of forces and moments exerted by the robotic end-effector. This development has become an alternative solution to the existing multi-axis load cell based on static force and moment sensors. The second one shows an experimental investigation on the performance of four different adaptive nonlinear *ℋ*_∞_ control methods applied to a constrained manipulator subject to uncertainties in the model and external disturbances. Coordinated position and force control is evaluated. Adaptive procedures are based on neural networks and fuzzy systems applied in two different modeling strategies. The first modeling strategy requires a well-known nominal model for the robot, so that the intelligent systems are applied only to estimate the effects of uncertainties, unmodeled dynamics and external disturbances. The second strategy considers that the robot model is completely unknown and, therefore, intelligent systems are used to estimate these dynamics. A comparative study is conducted based on experimental implementations performed with an actual planar manipulator and with the dynamic force sensor developed for this purpose.

## 1. Introduction

The first robotic manipulators were developed in order to perform positioning tasks. They were then specifically designed to be robust enough so as to not be affected by external disturbances. This physical robustness of robot manipulators has enabled researchers to obtain accurate positioning systems based on simple control laws. Decades later, the popularization of industrial robotics has heightened researchers' interest in creating a much wider range of applications for robotic manipulators in various environments.

Nowadays, many applications demand robotic manipulators to perform tasks subject to force and motion constraints. For example, the process of milling a piece requires accurate incidence angles, paths, forces and moments exerted by the drill in the milled material. Additionally, in industrial assembly lines, the objects must be assembled along certain paths with predetermined forces and moments. In sheet metal cutting, the cutting angles, paths and forces exerted on the material are also important. Moreover, on surfaces where polishing disks must always be perpendicular to the surface being polished, predetermined force must be applied. Consequently, new concepts of position and force control for lighter and more flexible robots have been created [1,2].

The problem defined in these applications involves three stages: the approach phase, the impact moment and the sustained contact tracking. The approach phase has been addressed in many works and defines the problem of positioning the tool without, or before, touching the environment. The second phase requires controlling the initial impact and damping out the vibrations generated during the event. After the initial impact, sustained contact is desired in many operations. In these cases, not only the motion of the end-effector is required to follow a prescribed path, but also the force exerted by the end-effector is required to follow a predefined reference. In these constrained systems, forces and moments generated between the end-effector and the target must be controlled, rather than being treated as disturbances and rejected. Addressing manipulators subject to model uncertainties and disturbances, the work considered in this paper is concentrated on the sustained contact tracking phase of the problem.

The great majority of solutions presented in the literature for the problem of constrained systems control require knowing the forces and moments of interaction between the robot end-effector and the environment where it is acting. The problem of force control undertaken in this paper was also considered in [1,3–7]. However, only simulation results were presented.

As a differential and important contribution, this paper addresses an experimental investigation on robust force control as a result of the development of a modular sensor device. The proposed device is designed and built to measure dynamic forces and moments in three orthogonal axes based on unidirectional force sensor units. As a consequence of its independent architecture on the type of sensitive material, static or dynamic force sensors can be applied. Piezoelectric or piezoresistive force sensors are effective solutions for the applications involved in this study due to their inherent dynamic response characteristics.

This paper is organized as follows: next section presents preliminary concepts and relevant results found in the literature; Section 3 introduces the model description of the constrained robot manipulator; Section 4 presents the problem formulation; Section 5 describes the solutions for the nonlinear *ℋ*_∞_ control problems based on the linear parametrization property of the model, neural networks and fuzzy systems; Section 6 demonstrates the 3D dynamic force and moment sensor; and Section 7 presents the experimental results for a three-link manipulator.

## 2. Preliminary Concepts

The concept of stiffness control was introduced by Salisbury [8]. It is based on the resistance of the environment in which the robotic end-effector applies the force. The problem is modeled as a mass-spring system and this method made possible the simultaneous position/force control. However, it considers constant desired position and force. In many robotic applications, such as when milling a piece, the end-effector must follow a trajectory along the surface of an object while applying a desired force, which is not necessarily constant. In this case, the stiffness control application does not work properly.

To address this type of limitation, Raibert and Craig [9] partitioned the control problem into two subtasks: one task is for controlling the position trajectory and the other task for controlling the desired force. This approach has been evolutionary for controllers, as proposed by Paul *et al.* [10], and became the conceptual basis of the hybrid trajectory of position and force control currently found in the literature.

It was shown by McClamroch and Wang [11], that when a manipulator is in contact with a surface, the position degrees of freedom are reduced. In this case, force constraint is added to motion equations through Lagrange multipliers. Thus, the order of the state vector is reduced in the dynamic equations of the manipulator.

Since the phase of controlling robotic systems with restricted position and force has been overcome, researchers began to focus on variables that could degrade the stability of the proposed models. The load on the end-effector that can fluctuate while the manipulator performs various tasks, friction and parameter uncertainties, are some examples that have required much research effort [12–15]. However, only a few studies have dealt with adaptive and robust control of robotic systems subject to constraints [16–20]. Using the basis developed by [9,11], the work performed by Chang and Chen [21] submitted an adaptive controller with *ℋ*_∞_ robust performance criterion for robotic systems with position and force constraints.

On the development of adaptive robust controllers, neural networks, combined with a nonlinear *ℋ*_∞_ control law, were applied by Chang and Chen [22] to control robotic systems subject to parametric uncertainties and external disturbances. A smooth response was achieved with a simple and computationally efficient implementation. Neural networks were applied to estimate the unknown dynamics of the system, thereby not requiring mathematical modeling knowledge. A variable structure controller (VSC) is added to this formulation by Chang and Chen [23]. The inclusion of VSC in the control law weakens the hypothesis used by the authors in [22] that the estimation error should be integrable, and limits it to be only a state-dependent function. Following this approach, the authors in [24] developed an adaptive *ℋ*_∞_ controller based on fuzzy systems and VSC for robots with position and force constraints.

Karayiannidis and Doulgeri proposed adaptive controllers in [5,6] for force and position trajectory tracking in environments with little or no constraint knowledge. In these papers, the main purpose is to explore the workspace using measurements of position, speed and force in a robotic end-effector, to attenuate impacts caused by unknown environments. In order to improve estimates of these models, the use of a camera was proposed by Cheah *et al.* [4] to provide a better estimate of the contact constraint through computer vision. However, these works are more interested in exploring the environmental constraint, rather than a repetitive task control with great tracking precision.

To satisfy the need of measuring the forces and moments of interaction between the robot end-effector and the environment, devices known as multi-axis load cell or multi-axis force and moment sensor are used. There are several patents on devices whose purpose is to measure forces and moments in three axes.

**Figure 1.**
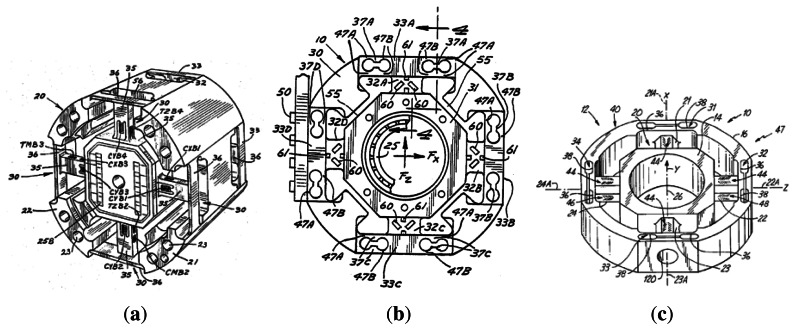
(**a**) Image taken from [25]; (**b**) Image taken from [26]; (**c**) Image taken from [27].

Force sensors, such as strain gages, have been generally used as the basic unit of measurement, as shown by the illustrative examples in Figure 1. The multi-axis load cell developed by Meyer and Lowe [25] was built in one piece with internal and external parts that are connected by a pair of axially spaced beams. The beams are fixed in the center of the piece, where the strain gages are attached, and an outside tunnel. The loads are measured by the curvature of the connectors. The load transducer developed by Meyer *et al.* [26] measures linear forces in three axes and moments of about two axes. The transducer has encapsulations connected by internal and external arms-sensitive loading. The device described by Sommerfeld *et al.* [27] consists of an external annular body, a hub and four beams that hold the hub to the radially outward portion. Strain gages are fixed to the faces of the beams, with a 90 degree lagging. Forces and moments exerted on the hub are transmitted to the four beams, and consequently, to the strain gages. Differently from [25,26], this device described by Sommerfeld *et al.* [27] is able to measure forces and moments in three orthogonal axes. However, all the devices mentioned above have an extrusion completely dependent upon the type of sensitive material being used.

## 3. Model Description

**Figure 2.**
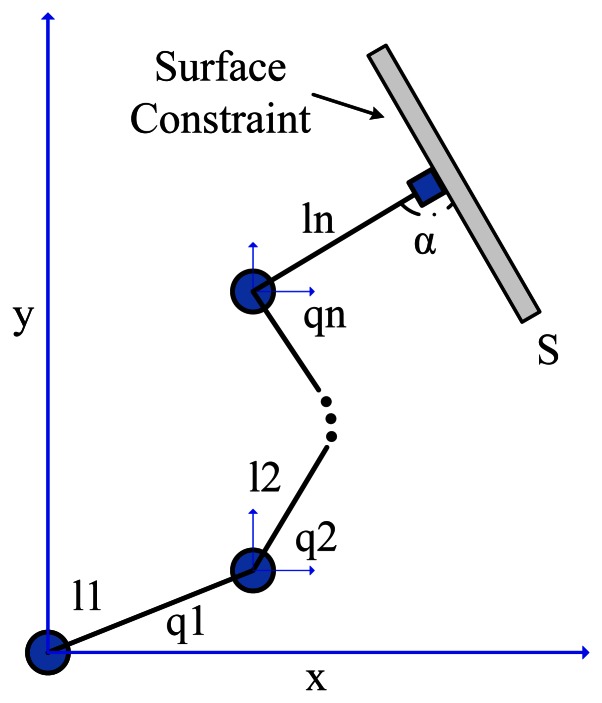
Constrained robot manipulator.

Let a constrained robot manipulator be defined by an n-link serial-chain rigid manipulator whose end-effector is in contact with a rigid environmental constraint, according to Figure 2.

The links of the manipulator are numbered from 1 to *n*: *J_i_* is the joint connecting the (*i* − 1)-th and *i*-th links, *l_i_* is the vector connecting *J_i_* and *J_i_*_+1_, *q_i_* is the angle of the *i*-th link around joint *J_i_*, *C_i_* is the center of mass of the *i*-th link and *l_ci_* is the vector connecting *J_i_* and *C_i_*. The constraint surface is represented by *S* and *α* is the angle of contact between the end-effector and *S*.

**Remark 1** *Assume that the end-effector is already in contact with the constraint surface, and the control exerted over the constraint force is such that the force will always maintain the end-effector in contact with the constraint surface*.

### 3.1. Robot Dynamics

The dynamic equations of a constrained robot is given from Lagrange theory as

(1) $$M(q)\ddot{q}+C(q,\dot{q})\dot{q}+G(q)=\tau +f+\tau_{d}$$

where *M*(*q*) ∈ ℜ^*n* × *n*^ is the symmetric positive definite inertia matrix, *C*(*q*, *q*˙) ∈ ℜ^*n* × *n*^ is the Coriolis and centripetal matrix, *G*(*q*) ∈ ℜ*^n^* is the vector of the gravitational torques, τ ∈ ℜ*^n^* is the torque vector acting upon the manipulator joint, *f* ∈ ℜ*^n^* denotes the vector of joint-space generalized forces on the environmental constraint exerted by the end-effector, and *τ_d_* defines finite energy unknown disturbances.

Model uncertainties in Equation (1) can be introduced dividing the matrices *M*(*q*), *C*(*q*, *q˙*), *G*(*q*), and *f* into a nominal and a perturbed part:

(2) $$\begin{array}{l} M(q)=M_{0}(q)+\Delta M(q)\\ C(q,\dot{q})=C_{0}(q,\dot{q})+\Delta C(q,\dot{q})\\ G(q)=G_{0}(q)+\Delta G(q)\\ f=f_{0}+\Delta f \end{array}$$

where *M*_0_(*q*), *C*_0_(*q*, *q*˙), *G*_0_(*q*), and *f*_0_ are nominal matrices and Δ*M*(*q*), Δ*C*(*q*, *q*˙), Δ*G*(*q*), and Δ*f* represent the uncertainties.

### 3.2. Constraint Modelling

Considering **Remark 1**, the *m*-dimensional surface constraint is described by the holonomic relationship

(3) ϕ(q)=0

where *ϕ*(*q*) : ℜ*^n^→* ℜ*^m^* is a smooth function.

Constraint forces are given by

(4) $$f=J_{c}^{T}(q)\lambda$$

where $J_{c}(q)=\frac{\delta \phi (q)}{\delta q}\in {ℜ}^{m\times n}$ is the Jacobian matrix that relates the constraint to the controlled variables of the robot and λ ∈ ℜ*^m^* is a vector of generalized Lagrangian multipliers associated with the constraint.

In this paper, it is considered that parametric uncertainties may also be included into the constraint model since the constraint surface may be not perfectly rigid, frictionless or even that its geometric description may not be exactly known. Thus, consider

(5) $$\begin{array}{l} \phi (q)=\Delta \phi (q)\\ J_{c}(q)=J_{c}(q)+\Delta J_{c}(q) \end{array}$$

and assume that Δ*ϕ* and Δ*J_c_* are implicit in Δ*f*, described in Equation (2).

### 3.3. Reduced Order Model

The presence of *m* constraints causes the manipulator to lose *m* degrees of freedom, and therefore, *n* – *m* linearly independent coordinates are sufficient to characterize the constrained movement. Therefore, to formulate a reduced order dynamics for the constrained system, the formulation presented in this paper follows the assumptions made by McClamroch and Wang [11] and posteriorly by Chang and Chen [24].

Define

$$q=\left[\begin{array}{c} q^{1}\\ q^{2} \end{array}\right]$$

where *q*^2^ = *σ*(*q*^1^) and *ϕ*(*q^1^*, *σ*(*q^1^*)) = 0.

The following reduced model formulation is obtained for the constrained manipulator as the model proposed by Chang and Chen [24]:

(6) $$A_{L}(q^{1}){\ddot{q}}^{1}+L^{T}(q^{1})C_{L}(q^{1},{\dot{q}}^{1}){\dot{q}}^{1}+L^{T}(q^{1})G(q^{1})=L^{T}(q^{1})(\tau +\tau_{d})$$

where

$$L(q^{1})=\left[\begin{array}{c} I_{\left(n-m\right)}\\ \frac{\partial \sigma (q^{1})}{\partial q^{1}} \end{array}\right]$$

and

$$C_{L}(q^{1},{\dot{q}}^{1}){\dot{q}}^{1}=M(q^{1})\dot{L}(q^{1})+C(q^{1},{\dot{q}}^{1})L(q^{1})$$

The necessary structure and properties of the model for controller formulation are maintained since *A_L_*(*q*^1^) = *L^T^*(*q^1^*)*M*(*q*^1^)*L*(*q*^1^) is symmetric positive definite and the matrix *A˙_L_*(*q*^1^) − 2*L^T^*(*q*^1^)*C_L_*(*q*^1^, *q˙*^1^) is skew symmetric.

## 4. Problem Formulation

Let *q_d_*(*t*) ∈ ℜ*^n^* and *q˙_d_*(*t*) ∈ ℜ*^n^* be the desired reference trajectory and the corresponding velocity for the joints, respectively Assume that *q_d_*(*t*) and its derivatives *q˙_d_*(*t*) and *q¨_d_*(*t*) are bounded. Define a bounded *f_d_* ∈ ℜ*^n^* as the desired reference contact force. To be consistent with the imposed restrictions, we need to assure that *ϕ*(*q_d_*) = 0 and $f_{d}=J_{c}^{T}(q_{d})\lambda_{d}$.

Since *q*^2^ = *σ*(*q*^1^), it is only necessary to find a control law that makes $q^{1}\to q_{d}^{1}$ when *t* → ∞. Therefore, define the position tracking error *x̄*_1_(*t*) and the filtered link tracking error *x̄*_2_(*t*), as in [24]:

(7) $$\bar{x}(t)\dot{=}\left[\begin{array}{c} {\bar{x}}_{1}(t)\\ {\bar{x}}_{2}(t) \end{array}\right]\dot{=}\left[\begin{array}{c} q^{1}(t)-q_{d}^{1}(t)\\ {\dot{q}}^{1}(t)-{\dot{q}}_{d}^{1}(t)+p(q^{1}(t)-q_{d}^{1}(t)) \end{array}\right]$$

for some constant *p* > 0.

From Equations (6) and (7), the error dynamic equations can be obtained as

(8) $$\dot{\bar{x}}=\left[\begin{array}{c} {\dot{\bar{x}}}_{1}\\ {\dot{\bar{x}}}_{2} \end{array}\right]=A\bar{x}+Bu+Bw$$

where

$$\begin{array}{c} \begin{array}{cc} A=\left[\begin{array}{cc} -pI & I\\ 0 & -A_{L}^{-1}(q^{1})C_{L}(q^{1},{\dot{q}}^{1}) \end{array}\right]\;, & B=\left[\begin{array}{c} 0\\ -{\left(M(q^{1})L(q^{1})\right)}^{-1} \end{array}\right] \end{array}\\ \begin{array}{cc} \begin{array}{cc} \text{u}=F\left(x_{e}\right)-\tau , & \omega =\tau_{\text{d}}, \end{array} & \text{and} \end{array}\\ F(x_{e})\dot{=}M(q^{1})L(q^{1})({\ddot{q}}_{d}^{1}-p{\dot{\bar{x}}}_{1})+C_{L}(q^{1},{\dot{q}}^{1})({\dot{q}}_{d}^{1}-p{\bar{x}}_{1})+G(q^{1}) \end{array}$$

Within this problem formulation, the torques applied to the joints to guarantee the task execution are given by

(9) $$\tau =F(x_{e})-u$$

where the term *F*(*x_e_*) refers to the dynamics of the controlled variables and *u* is the control law provided by the adaptive *ℋ*_∞_ controller proposed by Chang and Chen [24].

The term *F*(*x_e_*) can be divided in a nominal and uncertainty part, that is,

$$F(x_{e})=F_{0}(x_{e})+\Delta F(x_{e})$$

where term Δ*F*(*x_e_*) contains the uncertainties from parametric Equations (2) and (5).

In this paper, the control problem is solved based on two different approaches. In the first approach, an adaptive intelligent system is applied to estimate only the term Δ*F*(*x_e_*), considering that the nominal model of the robot is well known. In the second approach, it is considered that the system model or the term *F_0_*(*x_e_*) + Δ*F*(*x_e_*) is completely unknown, then the adaptive intelligent system is applied to estimate it. The nonlinear *ℋ_∞_* control and VSC are applied in both approaches to attenuate the effects of estimation errors and external disturbances.

## 5. Adaptive Nonlinear *ℋ_∞_* Controller Based on Intelligent Systems

The adaptive control laws presented as follows are based on two different learning methods to estimate uncertain parameters and also the behavior of unmodeled dynamics. Neural networks and fuzzy systems based on the Takagi–Sugeno model are considered. The structure of these intelligent systems can be seen in details in [28].

Let *F̂*(*x_e_*, Θ) = Ξ(*x_e_*)Θ be the output of the adaptive intelligent system, where *x_e_* is the input vector and Θ is a vector of adjustable parameters such that Θ*^T^*Θ ≤ *M_θ_*|*M_θ_* > 0. Two different cases are considered in the following:

Case 1 Estimation of Model Uncertainties Based on Intelligent Systems
  *In this case, the nominal model of the robot is considered as well known, the intelligent system then estimates only the model uncertainties, such as*
  $${\hat{F}}_{1}(x_{e},\Theta )\approx \Delta F(x_{e})$$
  *Therefore u and ω in Equation (8) will be rewritten as*
  $$\begin{array}{c} u=F_{0}(x_{e})+{\hat{F}}_{1}(x_{e},\Theta )-\tau \\ \omega =(\Delta F(x_{e})-{\hat{F}}_{1}(x_{e},\Theta ))+\tau_{d} \end{array}$$
  *so that ω includes the estimation error from the intelligent system*.
Case 2 Estimation of Complete Model Based on Intelligent Systems
  *In this case, however, the nominal model of the robot is considered completely unknown, so the intelligent system estimates the complete model, such as*
  $${\hat{F}}_{2}(x_{e},\Theta )\approx F_{0}(x_{e})+\Delta F(x_{e})$$
  *Therefore u and ω in Equation (8) is rewritten as*
  $$\begin{array}{c} u={\hat{F}}_{2}(x_{e},\Theta )-\tau \\ \omega =(F(x_{e})-{\hat{F}}_{2}(x_{e},\Theta ))+\tau_{d} \end{array}$$
  *so that ω includes the estimation error from the intelligent system*.

Regarding the nonlinear *ℋ*_∞_ control solution proposed by Chang and Chen [24] for constrained systems, define *u* = *ū* where *ū* = *u_P_* + *u_F_*, such that

(10) $$u_{P}=k_{0}E{\bar{x}}_{2}-k(x_{e})\text{sgn}(L{\bar{x}}_{2})$$

(11) $$u_{F}=J_{c}^{T}\lambda_{c}$$

where *u_P_* is the *ℋ*_∞_+VSC control term for the position enforcement and *u_F_* is the *ℋ_∞_* control law for the force tracking procedure, with

$$E:=\left[\begin{array}{c} I_{\left(n-m\right)}\\ 0_{m\times (n-m)} \end{array}\right]\;\text{and}\;\lambda_{c}\dot{=}\lambda_{d}-k_{\lambda }\int_{0}^{T}\left(\lambda -\lambda_{d}\right)dt$$

for some constant gain *k_0_, k*(*x_e_*) *>* 0 and *k*_λ_ > 0.

Theorem 1 presented in the following is a variation of the results presented in [21,23,24] with the difference that the nominal model and an intelligent system are considered rather than the linear parametrization of the robot. Thus, this theorem defines an adaptive *ℋ*_∞_ controller to solve the same problem's position/force tracking control problems presented in these papers but with the advantage of using the known model of the robot.

**Theorem 1** *Given a desired disturbance attenuation level γ > 0, a weighting matrix Q and the Lyapunov candidate function V(t), the following performance criterion*

(12) $$\begin{array}{cc} \int_{0}^{T}||\bar{x}(t){||}_{Q}^{2}\leq V(0)+\gamma^{2}\int_{0}^{T}||\omega (t)|{|}^{2}, & \forall \;T\geq 0 \end{array}$$

*is satisfied, for ω(t) ∈ L_2_ [0,∞), if there exists a dynamic state feedback controller*

(13) $$\dot{\Theta }=\{\begin{array}{ll} -\rho^{-T}\Xi^{T}L{\bar{x}}_{2} & \text{if}\;\left\|\Theta \right\|<M_{\theta }\;\text{or}\;(\left\|\Theta \right\|=M_{\theta }\;\text{and}\;{\bar{x}}_{2}^{T}L^{T}\Xi \Theta \geq 0)\\ -\rho^{-T}\Xi^{T}L{\bar{x}}_{2}+-\rho^{-T}\frac{{\bar{x}}_{2}^{T}L^{T}\Xi \Theta }{{\left\|\Theta \right\|}^{2}}\Theta & \text{if}\;\left\|\Theta \right\|=M_{\theta }\;\text{and}\;{\bar{x}}_{2}^{T}L^{T}\Xi \Theta <0 \end{array}$$

(14) $$\tau =F_{0}(x_{e})+\Xi \Theta -k_{0}E{\bar{x}}_{2}+k(x_{e})\text{sgn}(L{\bar{x}}_{2})-J^{T}\lambda_{c}$$

*which is the solution of the adaptive nonlinear ℋ_∞_ control problem subject to Equation (6) for the cases 1 and 2*.

The stability proof of this dynamic controller follows the line of the proof presented by Chang and Chen [24].

## 6. Force/Moment Sensor Development

The controllers proposed in Section 5 require the inferred force and moment measures in the robotic end-effector. The most accurate way to obtain such information is by measurements obtained through a multi-axes load cell.

**Figure 3.**
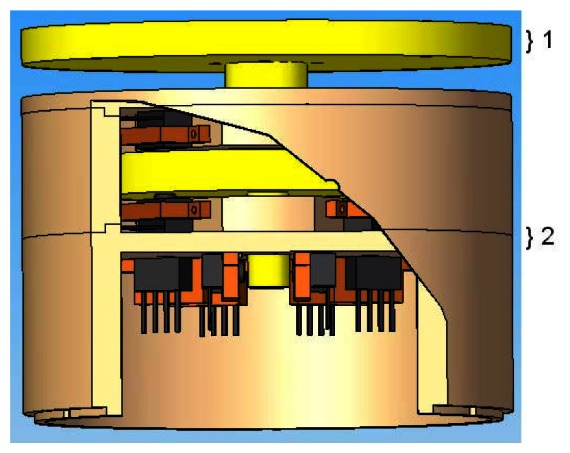
Design of the 3D dynamic forces/moments sensor: (1) Moving part; (2) Fixed part.

This section presents a detailed description on the modular 3D dynamic force/moment sensor device developed for this application purpose. Its modular architecture is shown in Figure 3.

### 6.1. Mechanical Description

**Figure 4.**
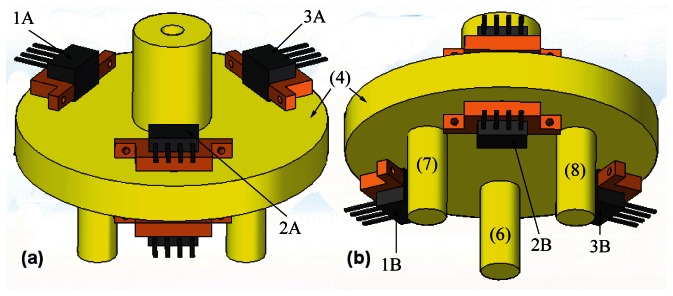
Body of force transmission (4) and arrangement of sensor units: (**a**) Top view; (**b**) Bottom view.

The 3D dynamic forces/moments sensor essentially comprised of two parts: a moving part and a fixed part. The moving part is shown in yellow in Figure 3(1). It is composed of a base (outside the sensor body) and a body of force transmission (inside the sensor body), which is responsible for transmitting forces and moments to the unidirectional force sensor units mounted on the fixed part, Figure 3(2). The device has an aluminum-made sensor board where twelve force sensor units are disposed in order to measure force and moment in all directions. The sensor units are arranged in pairs, for example, sensors 1A and 1B as shown in Figure 4, which illustrates the body of forces transmission (4) and identifies the sensor units.

**Figure 5.**
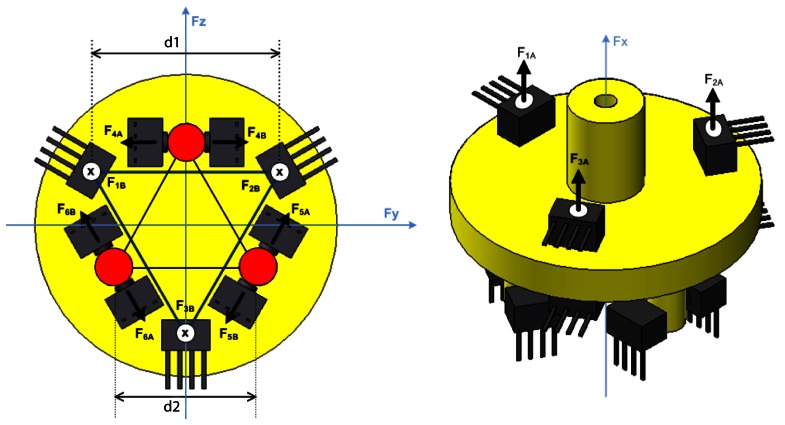
Schematic of sensor operation.

Each pair of sensor units measures the force in one direction. This setting is tensioned proportionally to the movement performed by (4) in that axis. Therefore, forces and moments normal to the plane of the base are calculated by the composition of measured forces in the three axes. Figure 5 demonstrates the sensor units operation schematic, and identifies the force measurements.

The formulation of the resultant forces/moments composition is obtained as follows.

Three pairs of sensor units, (1A,1B), (2A,2B) and (3A,3B), define, respectively, the force measurements (*F*_1A_,*F*_1B_), (*F*_2A_,*F*_2B_) and (*F*_3A_,*F*_3B_). The resultant forces of these three points can be expressed as

(15) $$F_{1}=F_{1A}-F_{1B}$$

(16) $$F_{2}=F_{2A}-F_{2B}$$

(17) $$F_{3}=F_{3A}-F_{3B}$$

It can be seen in Figure 5 that *F*_1_, *F*_2_, and *F*_3_ form an equilateral triangle, whose side is given by *d*_1_. In [29], Doebelin demonstrated that if three load cells, measuring force in one direction, are arranged triangularly as the vertices of the triangle (the measurement direction normal to the plane formed by the triangle), the following physical quantities can be calculated:

(18) $$F_{x}=F_{1}+F_{2}+F_{3}$$

(19) $$M_{y}=d_{1}\frac{F_{1}-F_{2}-(F_{3}-F_{1})}{2\sqrt{3}}$$

(20) $$M_{z}=\frac{d1(F_{3}-F_{2})}{2}$$

where *F_x_*, *M_y_*, and *M_z_* are the force along x-axis and the moments along axes y and z, respectively.

**Figure 6.**
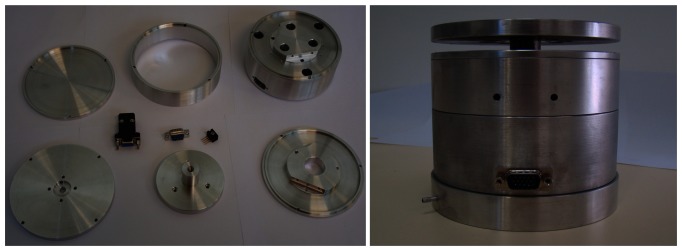
3D dynamic forces/moments sensor.

In the same way, *F*_4_, *F*_5_, and *F*_6_ also form an equilateral triangle, whose side is given by *d*_2_. Thus, the resultant forces of these three points can be expressed as

(21) $$F_{4}=F_{4A}-F_{4B}$$

(22) $$F_{5}=F_{5A}-F_{5B}$$

(23) $$F_{6}=F_{6A}-F_{6B}$$

and therefore,

(24) $$F_{y}=\frac{F_{5}-F_{4}-(F_{4}-F_{6})}{2}$$

(25) $$F_{z}=\frac{\sqrt{3}(F_{5}-F_{6})}{2}$$

(26) $$M_{x}=\frac{-d_{2}(F_{4}+F_{5}+F_{6})}{2\sqrt{3}}$$

where *F_y_*, *F_z_*, and *M_x_* are the forces along axes y and z and the moment along axis x, respectively. The sensor parts and the assembled device is shown in Figure 6.

The applied sensor unit provides a measurement range of 0 to 1.5 kgf, or a maximum load of 14.7 N. By means of Equations (18)–(20) and (24)–(26), the built sensor presents measurements characteristics as shown in Table 1.

### 6.2. Electronic Description

**Figure 7.**
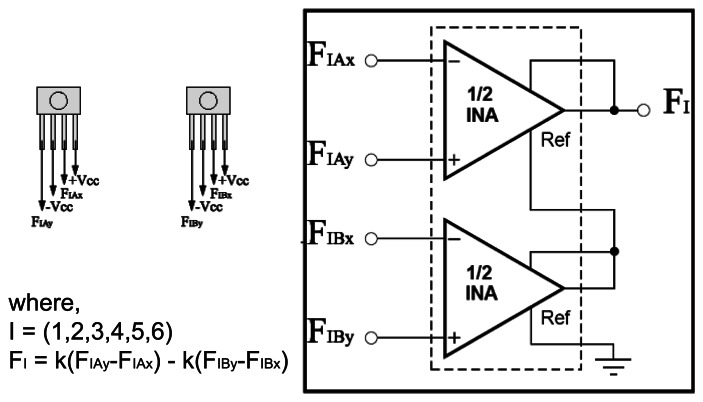
Electronic diagram.

The signal from each pair of unidirectional sensors are conditioned by an electronic circuit based on Instrumentation Amplifiers (INA). The electrical schematic of the signal conditioning circuit is summarized in Figure 7.

The sensors are powered by a symmetric supply (+ Vcc and −Vcc) and provide differential output signals (for example, *F*_1*Ax*_ and *F*_1*Ay*_).

Six INA comprise the printed circuit board (PCB). Each INA is responsible for conditioning output signals from a pair of force sensors. With this configuration it became possible to measure the differential forces applied on the moving part of the proposed device. The resultant force is then given by the following equation as described in Figure 7.

$$F_{I}=kF_{IA}-kF_{IB}$$

The signals generated by the INAs are directed to a data acquisition board that interfaces with the computer.

## 7. Experimental Results

**Figure 8.**
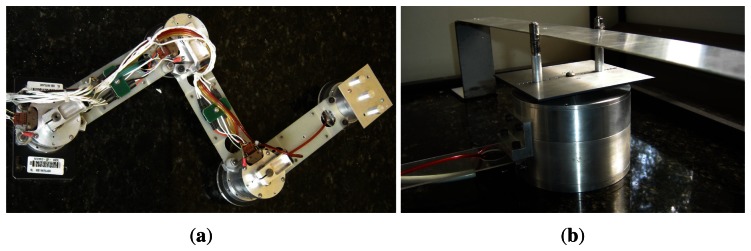
(**a**) UnderActuated Robot Manipulator; (**b**) Force sensor device coupled to the UARM end-effector.

The designed 3D dynamic forces/moments sensor operation is validated in a set of experimental applications, where a three-link planar manipulator is subject to a holonomic constraint defined by a straight rule. The proposed intelligent adaptive robust controllers are applied to this plant for position and force trajectory tracking. The experimental manipulator UARM (UnderActuated Robot Manipulator), whose nominal parameters are given in Table 2, is composed of a DC motor in each joint, a break and an optical encoder with quadrature decoding used to measure joint positions, Figure 8(a). Joint velocities are obtained by numerical differentiation and filtering [30,31]. Modeling matrices for the UARM, *M*(*q*), *C*(*q*, *q*˙), and *G*(*q*), can be seen in [30]. In Figure 8(b), the designed force sensor is coupled to the UARM end-effector which is constrained in a straight rule. A graphical user interface, whose software was developed using MATLAB^®^ platform, is used to implement control laws and interface with the experimental setup. It was implemented in a modular form, since any new controller can be easily developed and applied.

### 7.1. Implementation of Control Law

The constraint surface for the robot end-effector is a segment of a straight line on the X–Y plane. The angle *α* between the end-effector and the constraint line is defined in this application example as *π*/2. It means that the orientation must remain in a constant value *c* given through the line inclination *β* and *α,* where *c* = *π* + *atan*(*β*) − *α*. Hence, the equation of the *m* = 2 constraints is given by

$$\begin{array}{cc} \phi (q)=\left[\begin{array}{c} -l_{1}s_{1}-l_{2}s_{12}-l_{3}s_{123}+\beta [l_{1}c_{1}+l_{2}c_{12}+l_{3}c_{123}]+b\\ q_{1}+q_{2}+q_{3}-c \end{array}\right]= & \;[\begin{array}{c} 0\\ 0 \end{array}] \end{array}$$

where *b* is the linear coefficient of the constraint line, *s_ij_* = *sin*(*q_i_* + *q_j_*), *c_ij_* = *cos*(*q_i_* + *q_j_*). Hence, *ϕ*: ℜ^3^*→* ℜ^2^, and the Jacobian matrix, *J_c_*(*q*) = *∂ϕ*/*∂q*, is given by

$$J_{c}=\left[\begin{array}{ccc} J_{c11} & J_{c12} & J_{c13}\\ J_{c21} & J_{c22} & J_{c23} \end{array}\right]$$

with

$$\begin{array}{lll} J_{c11} & = & l_{1}c_{1}+l_{2}c_{12}+l_{3}c_{123}+\beta [l_{1}s_{1}+l_{2}s_{12}+l_{3}s_{123}]\\ J_{c12} & = & l_{2}c_{12}+l_{3}c_{123}+\beta [l_{2}s_{12}+l_{3}s_{123}]\\ J_{c13} & = & l_{3}c_{123}+\beta [l_{3}s_{123}]\\ J_{c21} & = & J_{c22}=J_{c23}=1 \end{array}$$

Defining *q^1^* = [*q*_1_] and *q*^2^ = [*q*_2_ *q*_3_], the matrix *L*(*q*) of the constraint line is

$$L(q)=\left[\begin{array}{c} 1\\ -\frac{\left[l_{1}\cos(q_{1})+l_{2}\cos(q_{1}+q_{2})+\beta (l_{1}\sin(q_{1})+l_{2}\sin(q_{1}+q_{2}))\right]}{l_{2}[\cos(q_{1}+q_{2})+\beta \sin(q_{1}+q_{2})]}\\ \frac{\left[l_{1}\cos(q_{1})+l_{2}\cos(q_{1}+q_{2})+\beta (l_{1}\sin(q_{1})+l_{2}\sin(q_{1}+q_{2}))\right]}{l_{2}[\cos(q_{1}+q_{2})+\beta \sin(q_{1}+q_{2})]}-1 \end{array}\right]$$

Initial and final coordinates of the movement are (*x*_0_,*y*_0_) = (0.46,0.38) m and (*x*(*T*),*y*(*T*)) = (0.53, 0.13) m, respectively. In this case, *β* = −3.57, *b* = 2.02, and *c* = 15.6°. The reference trajectory for the joint variables $q_{d}(t)=q_{d}^{1}(t)$ is a fifth-degree polynomial, with trajectory duration time *T* = 4 s. It is desired that no force acts on the normal direction of the constrain line and no moment acts on the *z*-direction, that is, λ*_d_* = [(*F_x_*)*_d_* (*M_z_*)*_d_*]*^T^* = [0 0]*^T^*.

We use as benchmarking for comparison of the proposed controllers, a controller with no intelligent adjust adaptation, that is, only the nominal parameters are used. In other words, we use the controller of Equation (14) without the ΞΘ term.

During the experiment, a limited disturbance was introduced at *t_i_* = 1.0*s* in the following form:

$$\tau_{d}=\left[\begin{array}{c} 0,01e^{\frac{-{\left(t-t_{d}\right)}^{2}}{2\mu^{2}}}\text{sen}(3,6\pi t)\\ -0,01e^{\frac{-{\left(t-t_{d}\right)}^{2}}{2\mu^{2}}}\text{sen}(2,7\mathrm{\pi t})\\ 0,01e^{\frac{-{\left(t-t_{d}\right)}^{2}}{2\mu^{2}}}\text{sen}(1,8\pi t) \end{array}\right]$$

**Figure 9.**
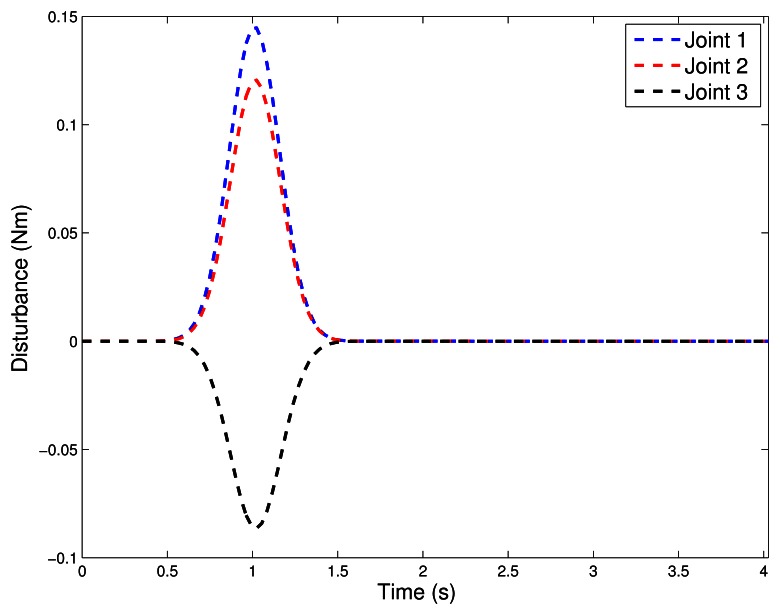
Torque disturbance.

If compared with the nominal torque, the disturbance *τ_d_* is approximately 64% of its peak value, see Figure 9.

The selected gains are defined in Table 3.

#### 7.1.1. Neural Networks Configuration

For the nonlinear *ℋ*_∞_ controllers via neural network, Cases 1 and 2, proposed in Section 5, let *n* = 3 be the number of joints of manipulator. Define

$${\hat{F}}_{\text{NNi}}(x_{e},\Theta )={\left[{\hat{F}}_{{\text{NNi}}_{1}}(x_{e},\Theta_{1})\;{\hat{F}}_{{\text{NNi}}_{2}}(x_{e},\Theta_{2})\;{\hat{F}}_{{\text{NNi}}_{3}}(x_{e},\Theta_{3})\right]}^{T}=\Xi \Theta$$

with *p*_1_ = *p*_2_ = *p*_3_ = 7 neurons in the hidden layer and the bias vector *b*_1_ = *b*_2_= *b*_3_ = [−3 −2 −1 0 1 2 3]. Using the reduced model, the input vector *x_e_* is defined by $x_{e}=\left[\begin{array}{ccccc} q^{1} & {\dot{q}}^{1} & q_{d}^{1} & {\dot{q}}_{d}^{1} & {\ddot{q}}_{d}^{1} \end{array}\right]$. In order to provide an input related to the error values, the weighting matrix for the first layer of the neural networks is defined by $W_{i}^{1}=W_{i}^{2}=W_{i}^{3}=[W_{ij}^{1}]=[W_{ij}^{2}]=[W_{ij}^{3}]=[1\;1-1-1-1]$. The activation function of the neurons in the hidden layer is chosen as the hyperbolic tangent, *G*(.) = *tanh*(.). The uncertain vector Θ is defined as $\Theta ={\left[\begin{array}{ccc} \Theta_{1}^{T} & \Theta_{2}^{T} & \Theta_{3}^{T} \end{array}\right]}^{T}$ and the matrix $\Xi ={\left[\begin{array}{ccc} \xi_{1}^{T} & \xi_{2}^{T} & \xi_{3}^{T} \end{array}\right]}^{T}$ as Pazelli *et al.* and Siqueira *et al.* showed in [28,30]. Θ values are updated at each control iteration by the adaptive law given in Equation (13). The neural networks outputs are given by

$${\hat{F}}_{{\text{NNi}}_{k}}(x_{e},\Theta_{k})=\sum_{i=1}^{p_{k}}\theta_{ki}G\left(\sum_{j=1}^{q_{k}}w_{\text{ij}}^{k}x_{ej}+b_{i}^{k}\right)=\xi_{k}^{T}\Theta_{k}$$

**Figure 10.**
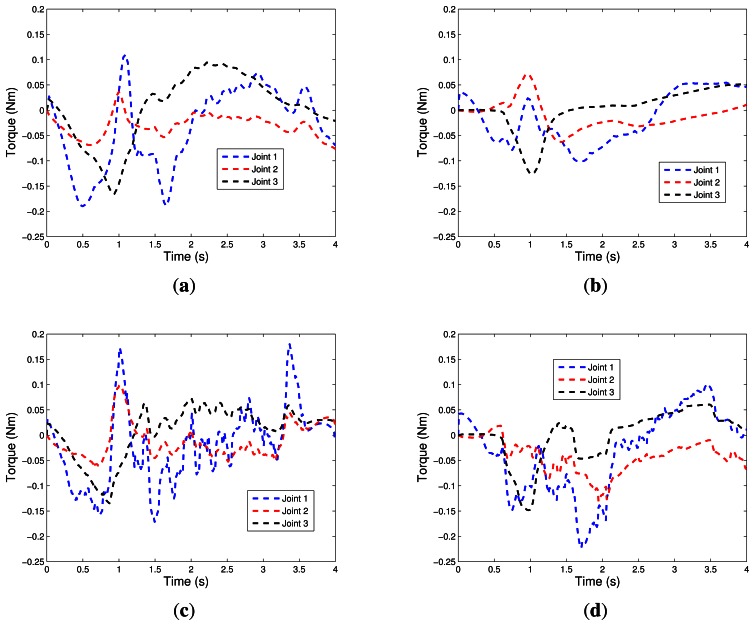

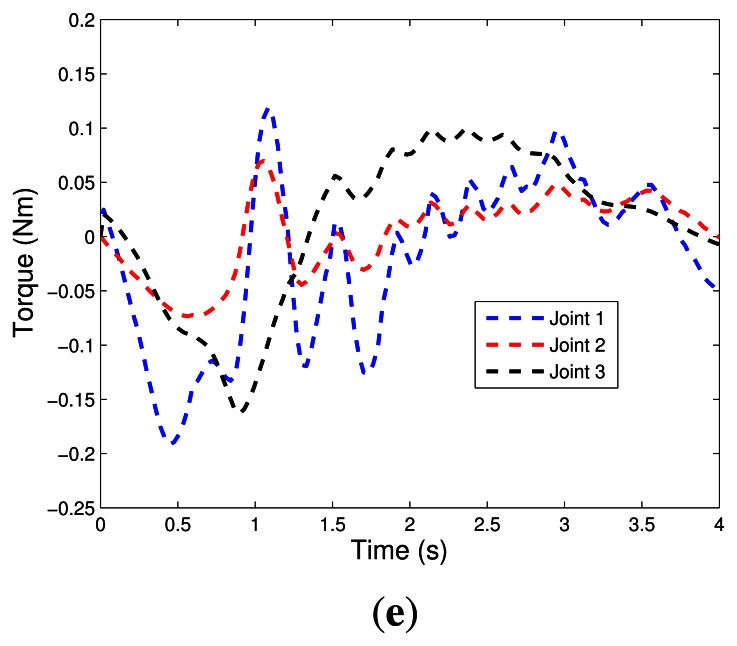
Joint Torque. (**a**) NN1; (**b**) NN2; (**c**) FS1; (**d**) FS2; (**e**) NOM.

**Figure 11.**
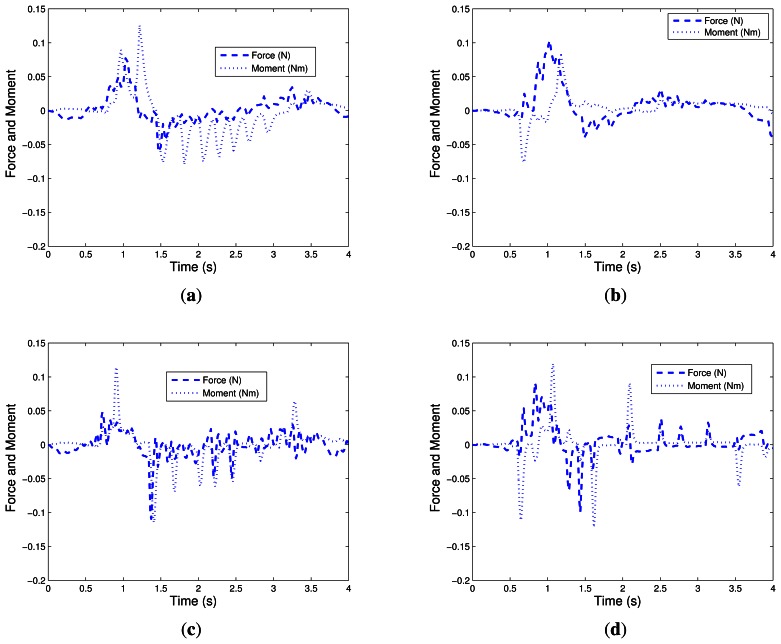

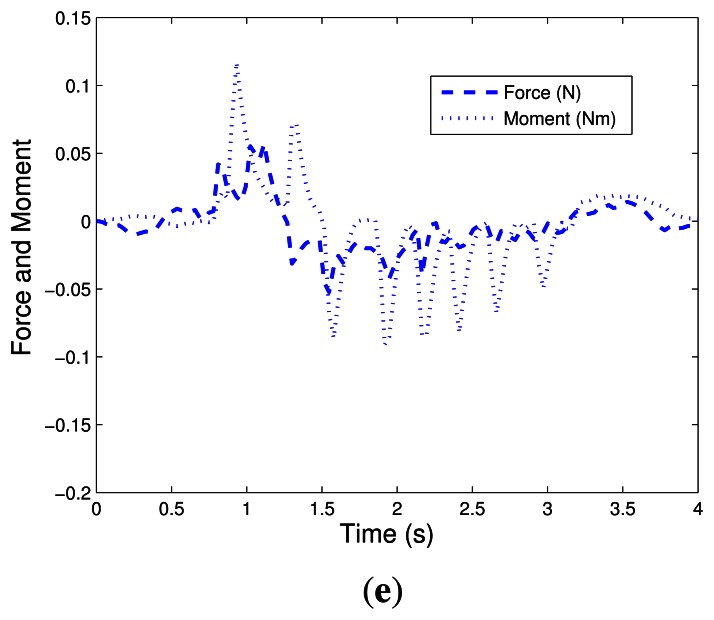
Force and Moment. (**a**) NN1; (**b**) NN2; (**c**) FS1; (**d**) FS2; (**e**) NOM.

**Figure 12.**
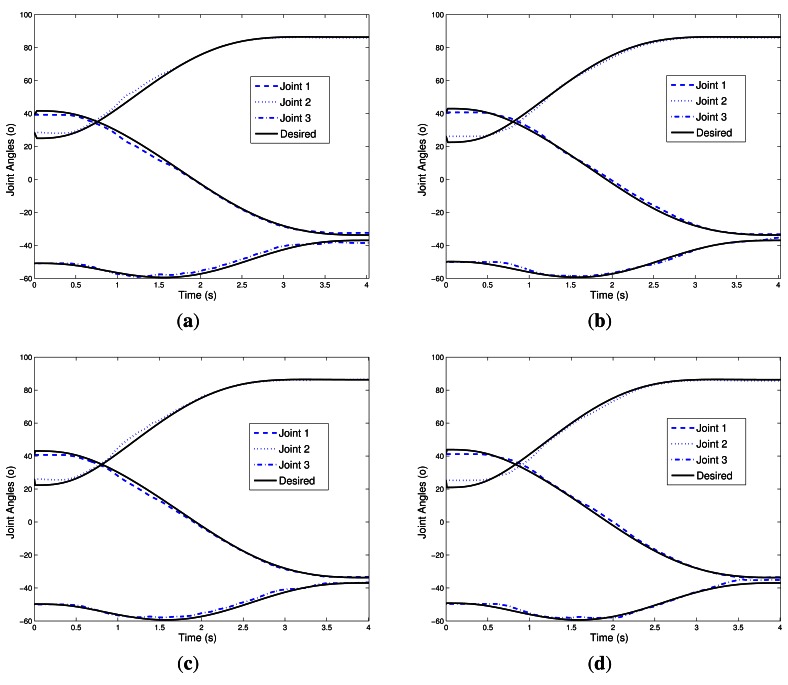

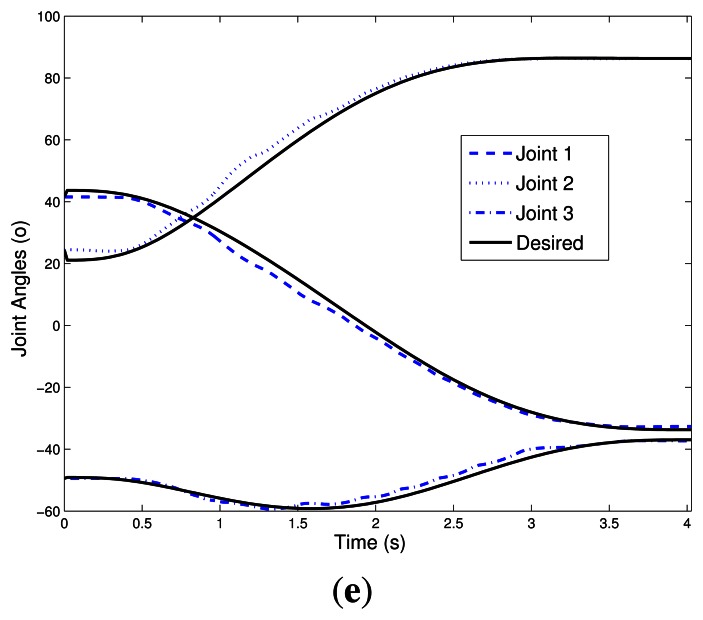
Joint Angles. (**a**) NN1; (**b**) NN2; (**c**) FS1; (**d**) FS2; (**e**) NOM.

Experimental results for the Case 1 (NN1) with neural network are shown in Figures 10(a), 11(a), and 12(a). For the Case 2 with neural network plus nominal model (NN2), experimental results are shown in Figures 10(b), 11(b), and 12(b).

**Remark 2** *Although NN1 and NN2 use the same structure and the same input data, their outputs are different. The online adaptation law provides the update of Θ values in distinct ways for each case. The contribution of NN1 output to the final torque signal is lower than the contribution of NN2 output, since NN1 estimates the value of ΔF(x_e_) and NN2 estimates the value of F(x_e_) + ΔF(x_e_). Thus, Θ values must achieve that requirement for each case*.

#### 7.1.2. Fuzzy Systems Configuration

**Figure 13.**
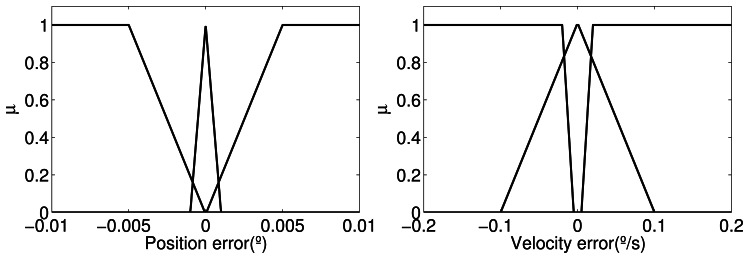
Fuzzy sets *A*_1_(*q̃*_1_) and *A_2_*(*q̃̇*_1_).

For the proposed adaptive fuzzy nonlinear *ℋ*_∞_ controllers, Cases 1 and 2 (Section 5), a set of *n* = 3 fuzzy systems may be defined by

$${\hat{F}}_{F\text{Si}}(x_{e},\Theta )={\left[\begin{array}{ccc} {\hat{F}}_{F{\text{Si}}_{1}}(x_{e},\Theta_{1}) & {\hat{F}}_{F{\text{Si}}_{2}}(x_{e},\Theta_{2}) & {\hat{F}}_{F{\text{Si}}_{3}}(x_{e},\Theta_{3}) \end{array}\right]}^{T}=\Xi \Theta$$

where *F̂_FSi_*_1_(.,.), *F̂_FSi_*_2_(.,.), and *F̂_FSi_*_3_(.,.) correspond to the estimate of the uncertain part of the dynamic behavior of joints 1, 2, and 3, respectively. The input vector *x_e_* is defined as $x_{e}=\left[\begin{array}{cc} {\tilde{q}}_{1} & {\dot{\tilde{q}}}_{1} \end{array}\right]$. The fuzzy sets $A(x_{e})=\left[A_{1}(\begin{array}{cc} {\tilde{q}}_{1}) & A_{2}({\dot{\tilde{q}}}_{1}) \end{array}\right]$ are defined according to Figure 13 and the number of linguistic variables in *U*_1_ and *U*_2_ are defined as *r*_1_ = *r*_2_ = 3. They are applied to both *F̂_FSi_*_1_(.,.), *F̂_FSi_*_2_(.,.) and *F̂_Fsi_*_3_(.,.) for the universe of discourse of position errors, $u_{2}^{1}=u_{1}^{2}=u_{1}^{3}={\tilde{q}}_{1}\in U_{1}^{1}=U_{1}^{2}=U_{1}^{3}=U_{1}$, and for the universe of discourse of velocity errors, $u_{2}^{1}=u_{2}^{2}=u_{2}^{3}={\dot{\tilde{q}}}_{1}\in U_{2}^{1}=U_{2}^{2}=U_{2}^{3}=U_{2}$.

During the control loop, these graphics are used to determine the grade of membership associated to *x_e_*, which defines the value of *μ* in the output of the Takagi–Sugeno fuzzy model; for more details see [28],

$${\hat{F}}_{F{\text{Si}}_{k}}(x_{e},\Theta_{k})=\frac{\sum_{i=1}^{p_{k}}\mu_{i}^{k}Y_{i}^{k}}{\sum_{i=1}^{p_{k}}\mu_{i}^{k}}=\frac{\sum_{i=1}^{p_{k}}\mu_{i}^{k}(\theta_{i0}^{k}+\theta_{i1}^{k}u_{1}+\theta_{i2}^{k}u_{2})}{\sum_{i=1}^{p_{k}}\mu_{i}^{k}}=\xi_{k}^{T}\Theta_{k}$$

A fuzzy rule base is defined with *p_k_* = *r*_1_*r*_2_ = 9 rules as

$$R_{i}:\text{IF}(u_{1}\;\text{is}\;A_{r_{1i^{1}}})\;\text{and}\;(u_{2}\;\text{is}\;A_{r_{2i^{2}}})\;\text{THEN}\;Y_{i}$$

The vector of adjustable components Θ is defined as $\Theta ={\left[\Theta_{1}^{T}\Theta_{2}^{T}\Theta_{3}^{T}\right]}^{T}$, with

$$\begin{array}{ccc} \Theta_{1}^{T} & = & \left[\theta_{10}^{1}\;\theta_{11}^{1}\;\theta_{12}^{1}\cdots \theta_{90}^{1}\;\theta_{91}^{1}\;\theta_{92}^{1}\right]\\ \Theta_{2}^{T} & = & \left[\theta_{10}^{2}\;\theta_{11}^{2}\;\theta_{12}^{2}\cdots \theta_{90}^{2}\;\theta_{91}^{2}\;\theta_{92}^{2}\right]\\ \Theta_{3}^{T} & = & \left[\theta_{10}^{3}\;\theta_{11}^{3}\;\theta_{12}^{3}\cdots \theta_{90}^{3}\;\theta_{91}^{3}\;\theta_{92}^{3}\right] \end{array}$$

and are updated at each control iteration by the adaptive law in Equation (13).

The matrix $\Xi ={\left[\xi_{1}^{T}\;\xi_{2}^{T}\;\xi_{3}^{T}\right]}^{T}$ is computed with

$$\begin{array}{ccc} \xi_{1}^{T} & = & \left[\xi_{10}^{1}\;\xi_{11}^{1}\;\xi_{12}^{1}\cdots \xi_{90}^{1}\;\xi_{91}^{1}\;\xi_{92}^{1}\right]\\ \xi_{2}^{T} & = & \left[\xi_{10}^{2}\;\xi_{11}^{2}\;\xi_{12}^{2}\cdots \xi_{90}^{2}\;\xi_{91}^{2}\;\xi_{92}^{2}\right]\\ \xi_{3}^{T} & = & \left[\xi_{10}^{3}\;\xi_{11}^{3}\;\xi_{12}^{3}\cdots \xi_{90}^{3}\;\xi_{91}^{3}\;\xi_{92}^{3}\right] \end{array}$$

Figures 10(c), 11(c), and 12(c) show the experimental results for Case 3. Figures 10(d), 11(d), and 12(d) show the experimental results of fuzzy system plus nominal model for Case 4.

### 7.2. Results Discussion

Figure 10 shows the applied torques in the robotic manipulator joints. Note that for the range of the disturbance inserted, the controllers act strongly on the system, reversing the directions of the torques of joints 1 and 2 and increasing the torque of joint 3. In this analysis, it appears that the controllers using neural networks, NN1 and NN2, oscillate less, therefore, they have a slightly slower response time.

The charts of Figure 11 are important because they show the behavior of the end effector during the controller actions, and represent the measurements of force and moment in the robotic end-effector. As expected, it was observed that during the period in which the disturbance appeared in the system, there was a higher intensity of forces and moments which decreased gradually, tending to zero, until the final time (4 s). The proposed configuration for the device sensor proves its effectiveness in providing these data with the required accuracy and without delay to the implementation of the control laws under study.

Figure 12 shows the tracking angles of three joints of the robotic manipulator. Notice that the largest deviation is in the range where the disturbance appeared. Nonetheless, the experiment showed good tracking of the trajectories desired. Again, we can see that for the controller without the intelligent system, NOM, the control actuation is slower, providing greater tracking error of reference signals.

Three performance indexes are used to compare the nonlinear *ℋ*_∞_ controllers: the *ℒ*_2_ norm of the state vector

$${ℒ}_{2}[\tilde{x}]={\left(\frac{1}{\left(t_{r}-t_{0}\right)}\int_{t_{0}}^{t_{r}}\left||\tilde{x}(t\right)|{|}_{2}^{2}dt\right)}^{1/2}$$

where ‖ · ‖_2_ is the Euclidean norm, the sum of the applied torques

$$E[\tau ]=\sum_{i=1}^{3}\left(\int_{t_{0}}^{t_{r}}\left|\tau_{i}(t)\right|dt\right)$$

and the sum of areas of contact forces

$$E[\lambda ]=\sum_{i=1}^{3}\left(\int_{t_{0}}^{t_{r}}\left|\lambda_{i}(t)\right|dt\right)$$

where λ*_i_*(*t*) the ith component of the contact forces. As the desired values of contact forces are zero, the lower the value of *E* [λ], the better the controller will be with respect to contact force control.

The results that are shown in Table 4 present the mean value of five experiments.

From Table 4 we can conclude that the controller based only on the nominal model presents a higher state error and greater forces. Although the fuzzy controllers exhibit the best performance, this performance difference with respect to controllers based on neural networks deserves more in-depth study, which will be carried out in future works.

## 8. Conclusions

In this work, the control problem of trajectory tracking with *ℋ*_∞_ guaranteed performance was considered for manipulators with force and position constraint. Five controllers were evaluated:

- The first controller, called nominal, considers that the term *F*(*x_e_*) is completely known, *i.e.*, it does not take into account parametric uncertainties of the model.
- The second and third controllers have neural network-based estimators, where the second considers the known nominal model of the manipulator and estimates only parameter uncertainties, and the third estimates the full model.
- The fourth and fifth controllers have fuzzy logic-based estimators which estimate only parametric uncertainties and the full model, respectively.

According to experimental results presented in Section 7, controllers NN1 and FS1 have better tracking trajectory compared, respectively, with the NN2 and FS2. Moreover, it also appears they are more stable because of the lower oscillations after the introduction of disturbance, and this feature occurs for the trajectory tracking in Cartesian space in order to follow the trajectory of joint angles. Comparing the results of intelligent systems it is noted that the controllers via neural networks (NN2 and NN1) have a smoother control action and, consequently, have smaller oscillations than the fuzzy systems (FS1 and FS2), which can be verified by comparing the graphics of torque (Figure 10). On the other hand, the controllers NN1 and NN2 are slower than the FS1 and FS2, hence they tend to have a greater tracking error. In addition, when the system is subject to disturbances, all controllers respond well.

Analyzing the proposed performance indices, it was shown that the state error in the fuzzy system-based controllers (FS1 and FS2) tend to be smaller than those based on neural networks (NN2 and NN1). A possible explanation for this is due to the fact that fuzzy systems acted faster than neural networks. It was observed that choosing values of *k*_λ_ more directly influence the adjustment of the forces, while the values of *ρ* influence the tracking errors in the state variables.

The development of the proposed sensor was of crucial importance for the realization of the experimental analysis, a differential of this paper. The measurement results shown proved the effectiveness of the proposed structure. Still, its modular feature enables the application of sensor units of various kinds, such as piezoelectric sensors.

As future work, a comparative study will be performed between the developed sensor and commercially available ones. Furthermore, the use of genetic algorithms to adjust controller gains is a current and ongoing project.

## Figures and Tables

**Table 1.** Maximum load.

	**Axis X**	**Axis Y**	**Axis Z**
Force (N)	44.1	29.4	25.5
Moment (N/m)	*d*_2_12.7	*d*_1_17	*d*_1_14.7

**Table 2.** UARM Parameters.

*Body*	*m_i_*	*I_i_*	*l_i_*	*lc_i_*

	(*kg*)	(*kgm*^2^)	(*m*)	(*m*)
Link 1	0.850	0.0075	0.203	0.096
Link 2	0.850	0.0075	0.203	0.096
Link 3	1.700	0.0900	0.240	0.177

**Table 3.** Selected Gains.

*P*	*k*_0_	*k*_λ_	*ρ*
$\left[\begin{array}{ccc} 2.50 & 0 & 0\\ 0 & 2.50 & 0\\ 0 & 0 & 2.50 \end{array}\right]$	$\left[\begin{array}{ccc} 0.35 & 0 & 0\\ 0 & 0.35 & 0\\ 0 & 0 & 0.35 \end{array}\right]$	$\left[\begin{array}{ccc} 2.00 & 0 & 0\\ 0 & 2.00 & 0\\ 0 & 0 & 2.00 \end{array}\right]$	$\left[\begin{array}{ccc} 0.75 & 0 & 0\\ 0 & 0.75 & 0\\ 0 & 0 & 0.75 \end{array}\right]$

**Table 4.** Performance Indexes.

	*ℒ*_2_[*x*]	*E*[*τ*]	*E*[λ]

	(*m*)	(*Nms*)	(*Ns*)
Nominal	0.1031	0.6508	0.1506
NN1	0.0780	0.6564	0.1435
NN2	0.1026	0.4318	0.1156
FS1	0.0759	0.5865	0.1136
FS2	0.0899	0.5182	0.1061
